# Validation of Internal Control Genes for Quantitative Real-Time PCR Gene Expression Analysis in *Morchella*

**DOI:** 10.3390/molecules23092331

**Published:** 2018-09-12

**Authors:** Qianqian Zhang, Wei Liu, Yingli Cai, A-Feng Lan, Yinbing Bian

**Affiliations:** 1 Institute of Applied Mycology, Plant Science and Technology College, Huazhong Agricultural University, Wuhan 430070, Hubei, China; qzhang196@163.com (Q.Z.); zhenpingliuwei@163.com (W.L.); loveylcai@163.com (Y.C.); 2 Key Laboratory of Agro-Microbial Resource Comprehensive Utilization, Ministry of Agriculture, Huazhong Agricultural University, Wuhan 430070, Hubei, China; 3 School of Biological Science and Engineering, Shanxi University of Technology, Hanzhong 723001, Shaanxi, China; lanafeng2004@126.com

**Keywords:** morel, qRT-PCR, gene expression analysis, housekeeping gene, normalizer genes

## Abstract

The reliability of qRT-PCR results depend on the stability of reference genes used for normalization, suggesting the necessity of identification of reference genes before gene expression analysis. Morels are edible mushrooms well-known across the world and highly prized by many culinary kitchens. Here, several candidate genes were selected and designed according to the *Morchella importuna* transcriptome data. The stability of the candidate genes was evaluated with geNorm and NormFinder under three different experimental conditions, and several genes with excellent stability were selected. The extensive adaptability of the selected genes was tested in ten *Morchella* species. Results from the three experimental conditions revealed that ACT1 and INTF7 were the most prominent genes in *Morchella*, CYC3 was the most stable gene in different development stages, INTF4/AEF3 were the top-ranked genes across carbon sources, while INTF3/CYC3 pair showed the robust stability for temperature stress treatment. We suggest using ACT1, AEF3, CYC3, INTF3, INTF4 and INTF7 as reference genes for gene expression analysis studies for any of the 10 *Morchella* strains tested in this study. The stability and practicality of the gene, vacuolar protein sorting (INTF3), vacuolar ATP synthase (INTF4) and14-3-3 protein (INTF7) involving the basic biological processes were validated for the first time as the candidate reference genes for quantitative PCR. Furthermore, the stability of the reference genes was found to vary under the three different experimental conditions, indicating the importance of identifying specific reference genes for particular conditions.

## 1. Introduction

True morels (*Morchella* spp.) belonging to *Ascomycota,* are consumed and appreciated worldwide due to their savory flavor and multiple bioactivities, including anti-oxidative [1,2], anti-inflammatory [3], antimicrobial [4], immunostimulatory [5] and antitumor properties [3,5,6,7]. In the 1980s and 90s, various studies were performed about morel in terms of physiology, ecology, cultivation, trophic type and classification [8,9,10,11,12]. However, little molecular biological research has been performed on morel, probably because its life cycle cannot be completed under the laboratory conditions [11,12,13,14]. On the basis of Ower’s pioneering study [10,15], *Morchella* mushrooms were realized of artificial cultivation in indoor and outdoor conditions [16,17], which gradually led to their commercialization in China since 2012 [14,18,19,20,21]. In 2017, the commercial cultivation area reached 4666 hectares in China. However, the lack of basic biology knowledge represents a high risk for commercial cultivation of *Morchella* mushrooms [14,20,21]. In recent years, the development of cultivation technology has promoted the improvement of biological research of *Morchella*, including developmental biology [20], population genetics [22,23], mating type [24,25,26] and senescence [18]. Differential gene expression in sclerotial formation of *Morchella conica* has been investigated [27], but no other reports were available about the molecular biology of *Morchella* spp. Fortunately, the transcriptome data could provide us a foundation for further molecular genetic research about morel. The lack of molecular insight of morels may be related to the failure to complete the whole life cycle under artificial conditions, and the progress of cultivation technology will promote its research on its molecular biology and genetics. Morel can be used as a model of edible epigeous macro-ascomycetes, which is conducive to the development of other ascomycetes, such as saddle mushroom (*Helvella* spp.), a well-known global species, which has also not been developed yet.

Gene expression analysis plays an important role in understanding the function of a specific gene [28]. Quantitative real-time PCR (qRT-PCR) is well-accepted as the most efficient method to evaluate gene expression [29], which combines PCR amplification and fluorescent signal detection for quantification of transcripts at an extremely low expression level. In the process of amplification, cycle numbers are recorded when the fluorescent signal intensity exceeds a specific threshold [30]. Thus, the cycle threshold value is used to describe the expression level of certain genes [31]. However, various factors, such as the quantity and quality of total RNA, reverse transcription efficiency, primer specificity, PCR condition and amplification efficiency, have huge effects on the accuracy of qRT-PCR results [32,33,34,35]. Among all these factors, the normalization of reference genes, which are defined as internal control genes or housekeeping genes used to normalize qRT-PCR expression analysis, is critical to minimize the variation or difference in the qRT-PCR results [36]. As such, inappropriate validation of reference genes can lead to enormous errors [35].

Recent studies have found that no single housekeeping gene is stably expressed across all experiments, especially in different conditions, organs or different tissues, demonstrating the necessity to identify specific reference genes for particular conditions [37,38,39]. In this study, we aimed to detect the housekeeping genes that showed relatively stable expression in morel under different conditions. Firstly, thirty-two genes of interest were selected and designed according to the transcriptome data of *M. importuna*. Then, the expression levels of all these genes were tested with fifteen samples distributed to three different experimental conditions. Next, all the candidate genes were ranked in stability using geNorm and NormFinder software. Finally, the most stable and general reference genes were evaluated for qRT-PCR normalization in different *Morchella* species.

## 2. Results

### 2.1. Primer Design and Verification

Based on the transcriptome sequencing results of *M. importuna* at different sclerotial developmental stages in our previous work, the reference genes expression primers were designed in this paper. Firstly, ten traditional reference genes of other crops were selected as candidate genes [39,40,41,42], such as lyceraldehyde-3-phosphate dehydrogenase (GPD), γ-actin (ACT), polyubiquitin (PUB), peptidyl-prolyl *cis-trans* isomerase (CYC), α-tubulin (ATU), elongation factor 1-α (AEF), HSP90 domain-containing protein (HSP90), Hsp70 protein-like protein (HSP70), and β-tubulin (BTU). Meanwhile, the relative expression of each gene at different developmental stages and the gene variation coefficient were obtained by quantitative analysis of the transcriptome data (All the transcriptome reads and final assembly results have been deposited at NCBI, under accession numbers SRR6655864-69 and GGGK00000000, respectively). The genes with the lowest variation coefficient were selected as candidate genes for further analysis [43]. A total of thirty-two reference genes were chosen and detected in this study. 

Specific primers were designed using the NCBI Primer-BLAST tool. The information about gene names and primer sequence is provided in Table 1. The amplification products were assayed by electrophoresis in a 1% agarose gel, and those with a specific single band at an expected length were selected, thus thirteen interested genes were excluded due to minor specificity (data not shown).

The mean amplification efficiency of the remaining genes was estimated by relative quantitative PCR with a standard curve of four serial 10-fold dilution of cDNA (ranging from 100 ng to 100 pg) for measuring the Cq value to a specific threshold [44]. The amplification efficiency was calculated by the formula: E = 10 ^(1/slope) [45]. For all the genes, the amplification efficiency values ranged from 93.3% to 107.9%, and the linear fit R^2^ was greater than 0.98, except CYC3 with a high value of 117.4%. Simultaneously, one single peak was required in real-time melting curve analysis of each gene. Finally, nineteen genes were selected as the candidate reference genes for further evaluation (Table 1).

### 2.2. Verification and Expression Stability of Candidate Reference Genes

**Figure 1:**
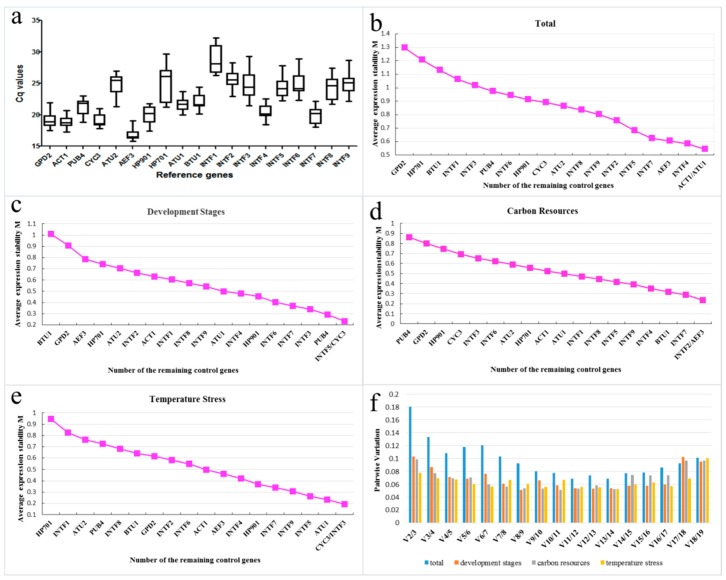
Expression stability analysis of the candidate reference genes in *Morchella importuna*. (**a**) Box plot graphs represent Cq values obtained from amplification curves. (**b**–**e**) expression stability values(M) calculated by geNorm; a lower M value indicates the most stable expression. (**b**) total datasets; (**c**) development stages; (**d**) carbon sources; (**e**) temperature stress. (**f**) pairwise variation (V) for the optimal number of reference genes in an accurate normalization. The pairwise variation (Vn/Vn+1) was analyzed between the normalization factors NFn and NFn+1 by geNorm.

Expression levels of the candidate genes for all the samples in different development stages, carbon sources, and temperature stress are presented in Figure 1. The Cq values of candidate genes varied obviously in the three different experimental conditions, ranging from 15.81 of AEF3 to 32.25 of INTF1 (Figure 1a). The stability of all the candidate genes was estimated by two Microsoft Excel-based tools. The nineteen genes in the fifteen samples were ranked using geNorm according to their expression stability measure M, based on the pairwise variation of a specific gene in all the reference genes. Consequently, the gene with the lowest M value was the most stable in expression [43], while the gene with the highest M value was poor in stability. The optimal number of reference genes for an accurate normalization was determined by the pairwise variation values (Vn/Vn+1) obtained by geNorm. 

A cut-off value of 0.15 was used by Vandesompele et al. below which the inclusion of an additional reference gene is not required [43]. Compared with geNorm, NormFinder adds intra and inter group variations to calculation of normalization factors, and different results are expected due to distinct statistical algorithms of geNorm and NormFinder [43,46].

When considering all the datasets, ACT1/ATU1 (M = 0.54) was the best pair as internal control genes, while GPD2 was the least stable gene (M = 1.29) (Figure 1b). However, INTF7/INTF4 was determined by NormFinder as the most ideal pair of reference genes. In both programs, HP701, BTU1 and GPD2 were ranked as the least stable genes (Table 2 and Table 3; Figure 1b).

For different development stages, INTF5/CYC3 (M = 0.23) was determined as the most suitable pair by geNorm, while INTF5 and CYC3 as the most stable and the third stable gene by NormFinder, respectively. GPD2, HP701 and BTU1 showed the least stability again (Table 2 and Table 3; Figure 1c), indicating that they were not fit for quantitative real-time PCR normalization in *M. importuna* under such conditions.

For different carbon sources, INTF2 and AEF3 had an equivalent M value of 0.23, and were defined as the most stable pair of genes by geNorm. However, INTF4 was identified as an ideal gene by NormFinder, followed by AEF3, while INTF2 was ranked as the fourth reliable gene (Table 2 and Table 3; Figure 1d). Meanwhile, CYC3 and ATU2 were excluded due to the lowest expression level in different carbon sources.

For temperature stress, CYC3/INTF3 showed the lowest M value of 0.19, followed by ATU1 (M = 0.23) whereas the ATU1/INTF3 pair showed the best performance as determined by NormFinder. In both geNorm and NormFinder software, INTF1 and HP701 showed the highest variation in all the internal control genes (Table 2 and Table 3; Figure 1e).

Moreover, the pairwise variation values obtained by geNorm for the least number of reference genes in an accurate normalization were also assessed. All the V values for each experimental set were below 0.15, indicating that there is no need to add a third gene as a reference gene. However, considering the total datasets, the optimum number of reference genes increased to three (Figure 1f).

### 2.3. Validation of the Selected Reference Genes in Morchella

**Figure 2:**
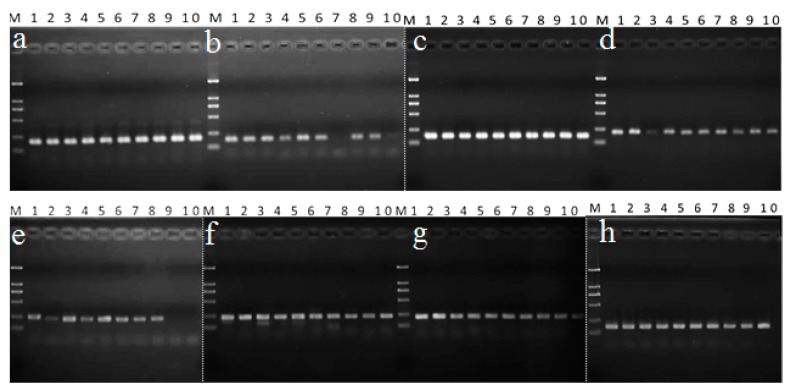
Validation of generality of the selected reference genes in different *Morchella* species. The order of the phylogenetic name of the species indicated as lanes 1–5 represent Mel-10, Mel-6, Mel-21, Mel-21/33 and Mel-13/26 belonging to Elata clade, while Lanes 6-10 represent Mes-23/24, Mes-6, Mes-21, Mes-19 and Mes-24 belonging to Esculenta clade. Selected reference genes: (**a**) ACT1; (**b**) ATU1; (**c**) AEF3; (**d**) CYC3; (**e**) INTF2; (**f**) INTF3; (**g**) INTF4; (**h**) INTF7; The gels of different batches were separated by the white dotted line.

The multigene phylogenetic analysis showed that there are about 68 phylogenetic species in *Morchella* all over the world, which belong to three branches: the basic branch Rufubrunnea clade, the sister branches Esculenta clade and Elata clade [14,47,48]. The basic branch Rufubrunnea clade contains two species, *Morchella anatolica* and *Morchella rufobrunnea*, which occurred only in North America and Europe [47]. The Esculenta and Elata clade formed a large group of 66 different species. Recent studies have shown that there are 30 *Morchella* phylogenetic species attributed to the Esculenta and Elata clades in China [49]. In order to validate the stability and generality of the selected reference genes in different development stages in *Morchella*, the CYC3 and INF5 primer pair was selected and evaluated. The amplification results revealed that CYC3 showed stable expression, but INTF5 was very unstable in all the ten morel species (Figure 2d).

The top-ranked reference genes for different carbon sources and temperature treatments were also validated. INTF4 and AEF3 performed well in different carbon sources while INTF2 only showed ideal stability among eight species (Figure 2c,g,e). Additionally, INTF3 and ATU1 were two top-ranked genes in temperature treatment (Figure 2f,b). INTF3 showed high consistency in all the species and ATU1 performed well except for two species in Esculenta (Mes-6 and Mes-24) (Table 4).

**Figure 3:**
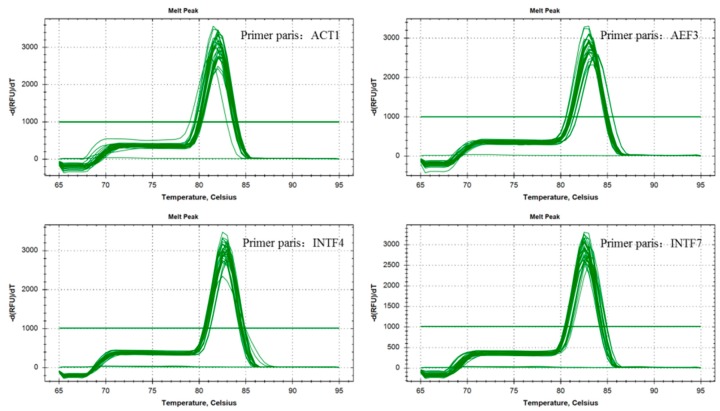
Amplification and dissolution curves of selected primers among ten different morel species.

Moreover, ACT1 and INTF7 were further examined due to their high stability in all the data (Figure 2a,h). In addition, the dissolution curves were used to confirm the amplification efficiency of primers in ten samples. The results showed that the selected primers (ACT1, AEF3, INTF4 and INTF7) had a single dissolution curve, and the results were consistent with the electrophoresis results (Figure 2a,c,g,h; Figure 3). The results confirmed that both of them could serve as suitable reference genes for normalization in the genus of *Morchella*.

### 2.4. qRT-PCR of Amylase Gene under Different Carbon Sources with Different Internal Control Genes

**Figure 4:**
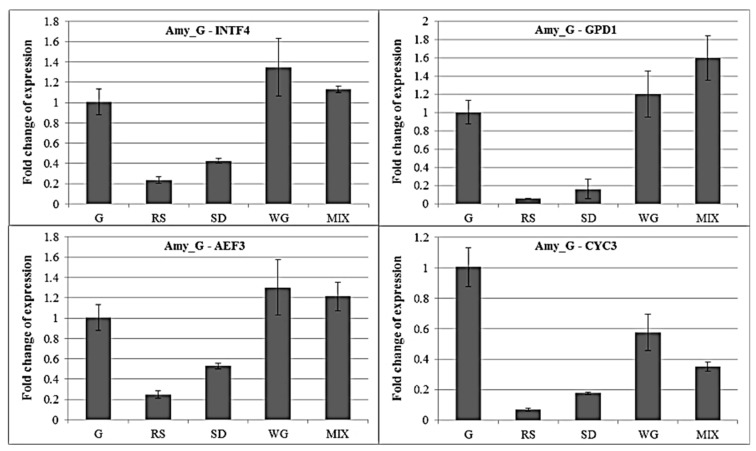
Effects of different normalizing genes on measured relative mRNA abundance. Hypothetical amylase gene (Amy_G) was used to quantify the expression of five different carbon sources (G: glucose, SD: sawdust, WG: wheat grain, RS: rice, MIX: containing equal proportions of rice straw, sawdust and wheat grain). The amount of expression in G was used as a control. Two stable (INTF4, AEF3) and unstable (GPD1, CYC3) internal control genes were used for analysis, respectively. Calculation of the fold change of expression by 2^−∆∆Ct^ methods [52].

To verify the importance of accurate normalization, RNA was prepared from five different carbon sources (G: glucose, SD: sawdust, WG: wheat grain, RS: rice, MIX: containing equal proportions of rice straw, sawdust and wheat grain as in materials and experimental treatments mentioned) and subjected to qRT-PCR for analysis of one hypothetical starch degradation gene. Data obtained was then normalized with both stable (INTF4/AEF3) and unstable (GPD1/CYC3) internal control genes, as suggested by the aforementioned results. As *M. importuna* has a good ability to degrade starch [14], the amylase genes, Amy_G (primer: GATTGGGAGCTTTGAGCAAG/GTGTTCTCTTCTGGGTTGGA, amplification product size: 185 bp, Tm: 60 °C, annotation as amylo-1,6-glucosidase compared to the Nr XP_007781986 with E-value = 0), was selected to analyze the expression levels of different carbon sources. As shown in Figure 4, two stable internal control genes INTF4 and AEF3 were selected for control, and the expression patterns of Amy_G gene in the five matrices were consistent, which expression in WG and MIX was higher than that in RSand SD, and is the lowest in RS (downregulation to 24% contrast to G) and the highest in WG (1.35 times upregulation contrast to G). However, when the unstable GPD1 or CYC3 was used as a control, the pattern of the Amy_G gene expression was different, which had the lowest in RS (downregulation to 5.8%) and the highest in MIX (1.6 times upregulation) under the GPD1 as control, and had the lowest in RS (downregulation to 7.0%) and the highest in WG (0.57 times) under the CYC3 as control. Selecting different reference genes as internal control, the maximum differential multiple of the Amy_G gene expression was 4.26 times (RS under AEF3 and GPD1 as control). It is thus obvious that normalization with different internal control genes would result in completely different conclusion, as also reported by others [50,51].

## 3. Discussion

In order to exclude variations caused by RNA quantity, integrity, reverse transcription efficiency and other factors [39], it is imperative to correct the expression of reference genes during qRT-PCR analysis. However, there is no universal reference gene whose expression level remains constant in all conditions, thus finding a particular reference gene for qRT-PCR in certain conditions is significant [53]. Traditional reference genes such as Actin, β-tublin, GAPDH, EF1α and 18S rRNA, which were broadly used as stable internal control genes in human and other model creatures in PCR reactions, were found not always stable in different organs or conditions [28,32,54,55,56]. The normalization of reference genes is really essential before quantitative real-time PCR in specific species or new research materials.

Identification and evaluation of reference genes have been performed in many species [28,32,34,35,40,41,54,57,58,59,60], but no such studies have been performed in *Morchella*, even in pezizales. In our previous work, the transcriptome of *Morchella importuna* No-4 strain was sequenced (unpublished), with a purpose to obtain several housekeeping genes in *Morchella* under different development stages, carbon sources and temperature stresses for qRT-PCR analysis. In this study, the candidate genes were evaluated using the two frequently used tools of geNorm [43] and NormFinder [46]. The expression stability of the nineteen target genes showed high variation in the three experimental sets. The performance of the prominent reference genes was found extremely inconsistent under different conditions, whereas the least stable genes could be circumvented by both programs. In this case, traditional reference genes such as GPD2, BTU1 and ATU were all excluded as unsuitable internal control genes. Additionally, HP701 and INTF1 were confirmed as variable genes under the temperature stress set. HP701 gene was annotated as Hsp70 protein-like protein, which was an important part of the cell’s machinery for protein folding and favorable to protect cells from stress [61]. INTF1 was annotated as similar to DNA damage response protein wss1 [62] (Table 1). Functionally, the expression stability of the HSP701 and INTF1 genes is more susceptible to temperature stress. Thereby, GPD2, BTU1, ATU2, INTF1 and HP701 were considered inappropriate for normalization of genes in *Morchella* under such a condition.

ACT1 and ATU1 were confirmed as the most suitable reference genes by geNorm among all the datasets, although they were not top-ranked by NormFinder (INTF7 was ranked as the best one, which was annotated as 14-3-3 protein, a family of conserved regulatory molecules and expressed in all eukaryotic cells [63]) in *M. importuna*. Actin (ACT1) was used as a universal reference gene in many crops. 14-3-3 protein (INTF7) has the ability to bind a multitude of functionally diverse signaling proteins, including kinases, phosphatases, and transmembrane receptors. Functionally, the INTF7 gene can be used as a stable reference gene in crops. α-Tubulin (ATU) was used as a universal reference gene in many fungi [64,65], but ATU1 was not the top preferred gene here. Based on the integrated data here and from other morel species, ACT1 and INTF7 were chosen as the prominent pair of genes for normalization in *Morchella*.

In different development stages, INTF5 (annotated as similar in function to ubiquitin carboxyl-terminal hydrolase 4) [66] and CYC3 were assumed to be the best pair of reference genes. Consistently, CYC3 revealed stable expression in all the ten morel species (Figure 2d). However, IINTF5 was found extremely reliable as an internal control gene in *M. importuna* but not in the other morel strains, probably due to the matching problem of primers.

For different carbon sources, AEF3/INTF2 and INTF4 primer pairs performed well as determined by both geNorm and NormFinder software. These three genes were also evaluated with all the ten morel strains. INTF2 was found unstable for Mes-19 and Mes-24. Elongation factor 1-alpha (AEF3) was commonly used as a universal reference gene in other crops [41,59]. Vacuolar ATP synthase (INTF4, annotated as vacuolar ATP synthase 16kDa proteolipid subunit) is required for an array of molecular processes, including protein sorting, zymogen activation, receptor-mediated endocytosis and synaptic vesicle proton gradient generation [67], but so far, no research has been performed on the reference gene for gene expression analysis. Accordingly, AEF3/INTF4 was determined as the best pair of reference genes for different carbon sources.

Under temperature stress, four top-ranked genes have similar stability by both geNorm and NormFinder software, but with a slight difference in position. CYC3/INTF3 was the best pair of reference genes by geNorm, while ATU1/INTF3 was the most ideal pair of reference genes by NormFinder. Besides, INTF5 was ranked the third by NormFinder and the fourth place by geNorm under temperature treatment. Therefore, INTF3 was determined by both two programs as a stable reference gene for normalization of genes under different temperature stresses. INTF3, vacuolar protein sorting/targeting protein 10, functions as a sorting receptor in the golgi compartment required for the intracellular sorting and delivery of soluble vacuolar proteins, which has reason to be a stable candidate gene based on its biological function of protein [68]. CYC3 appeared to be the most controversial gene with high stability in temperature stress and development stage tests, but exhibited the opposite results in the carbon source tests (Table 2 and Table 3). Therefore, CYC3 was suggested as an internal control gene in temperature stress and development stage tests in *Morchella*.

The optimal number of reference genes varied slightly between different experimental conditions and total database, and two reference genes were required for normalization of gene expression analysis in this paper.

In summary, the stability of reference genes was validated under different experimental conditions, demonstrating the importance of identification of specific reference genes prior to quantitative real-time PCR. Collectively, ACT1/INTF7, INTF4/AEF3, and INTF3/CYC3 were ranked as the most stable reference genes in *Morchella* based on the results from all the three experimental sets of different development stages, carbon sources and temperature stresses. This study has provided several ideal reference genes for further evaluation of quantitative real-time PCR gene expression analysis in *Morchella*.

## 4. Materials and Methods

### 4.1. Materials and Experimental Treatments

The commercial cultivation strain *Morchella importuna* No-4 was used in this study, which has been cultured in China for many years and stored in the Institute of Applied Mycology of Huazhong Agricultural University. The transcriptome of this strain at different sclerotial development stages has been sequenced and analyzed (data not shown), which allowed us to obtain a large number of candidate genes for the expression stability analysis. Fifteen samples of *M. importuna* were collected and divided into three experimental sets, including six development stages, six different carbon sources for mycelial growth and three temperature treatment sets.

To detect the gene expression during different development stages, six samples were obtained as mycelium, white sclerotia, mature sclerotia, primordium, small ascocarps and mature ascocarps. Furthermore, the mycelium of *M. importuna* was inoculated on six different carbon sources, including glucose, sawdust, wheat, rice, mix and a carbon-free CYM liquid medium, which were all with 2% final concentration improved on the basis of CYM liquid medium (yeast extracts 2 g L^−1^, peptone 2 g L^−1^, K_2_HPO_4_ 1 g L^−1^, MgSO_4_ 0.5 g L^−1^, KH_2_PO_4_ 0.46 g L^−1^). The glucose in CYM was separately replaced by sawdust, wheat and rice. The mix sample was a mixture of sawdust, wheat and rice with 0.67% final concentration for mycelium culture. The carbon source experiments were performed by incubation at 23 °C for seven days in the dark. The low and high temperature treatment sets were used as a temperature pressure test. The mycelia were incubated at 23 °C in CYM liquid medium for six days, then treated separately at 4 °C and 32 °C for 24 h, with untreated mycelium as a control. The as-treated mycelia were collected and rinsed in ddH_2_O, followed by dehydration through clean filter paper extrusion, freezing in liquid nitrogen and storage at −80 °C until RNA extraction.

### 4.2. Selection of Reference Genes and Primer Design

In our previous study, a transcriptome sequencing and de novo assembly work were conducted on *M. importuna* at different sclerotial development stages (under publication), thus enabling us to select and design the candidate reference genes in this paper. Meanwhile, the expression of the gene was analyzed (transcripts per million, TPM), and the variation coefficient was calculated for the transcript expression in the different stages [69]. According to the lowest coefficient of variation (CV < 0.08) and the size of expression abundance, the candidate genes were selected. The generally used housekeeping genes in other fungi, plants and animals were also employed based on the gene annotation results. The cDNA sequences of candidate genes were extracted, and the location information of introns and exons was determined through genome alignment from transcriptome to genome of *M. importuna* (genomic data has been submitted to the NCBI database, accession number: QOKS00000000). Primers were designed by using the NCBI primer-BLAST design tool (https://www.ncbi.nlm.nih.gov/tools/primer-blast) [70]. The primer design principle was as follows: the size of the target fragment was between 150–300 bp, the temperature was at 60 ± 3 °C, the optimal length of primer was 20 bp, and the primers could cross one intron at least.

The specificity of all the designed primers was verified through 1% agarose gel electrophoresis before qRT-PCR validation. The amplification reaction of each pair of primers was performed with 0.3 μL of 4 × dNTPmix, 0.3 μL of rTaq (Takara, Nojihigashi 7-4-38, Kusatsu, Japan), 2 μL 10 × PCR buffer and 10 nM each of primer in a total volume of 20 μL, under the conditions of 95 °C for 3 min, followed by 35 cycles of 95 °C for 15 s, 60 °C for 15 s and 72 °C for 15 s. The size of the primer amplification product was consistent with the target fragment, with a single and clear band present under agarose gel electrophoresis, and such primers were selected for subsequent analysis.

### 4.3. Total RNA Isolation and cDNA Synthesis

The total RNA was extracted from 100 mg of each sample powder in liquid nitrogen, using the phenol/SDS method. DEPC-treated water was mixed with 10 × STE buffer and 10% sodium dodecyl sulfate at a volume ratio of 7/2/1 to release the nucleic acid, then the nucleic acid was extracted with phenol solution (phenol/chloroform/isoamyl alcohol at a volume ratio of 25/24/1). Isopropanol alcohol was used to deposit the total RNA, then the RNA deposit was dissolved in DEPC water, the residual DNA was digested by DNAase I (Thermo Scientific, Waltham, MA, USA) to remove DNA contamination, and the final pure RNA solution was stored at −80 °C. RNA concentration and quantity were determined using a DS-11+Spectrophotometer (Applied Denovix, Wilmington, DE, USA), and the RNA samples with only one single peak at 260 nm and the OD260/OD280 ratio between 1.8 and 2.2 were used for further analysis. The integrity of RNA was also assessed by electrophoresis on 1% agarose gel. The cDNA strand was synthesized using HiScript II Reverse Transcriptase (Vazyme, Nanjing, China) with 500 ng of total RNA in 20 μL reaction volume according to the protocol of the manufacturer. The cDNA products were diluted 1:5 with double-distilled water prior to quantitative real-time PCR analysis.

### 4.4. Quantitative Real-Time PCR Analysis

The amplification of qRT-PCR was carried out in a 96-well optical plate with a CFX Connect Real-Time System (Applied with Bio-Rad, Hercules, CA, USA). The reaction system consisted of 5 μL of SYBR Green Master Mix (AceQ^TM^ qPCR, Vazyme), 2.5 nM of each primer, and 1 μL of diluted cDNA in a final volume of 10 μL. A two-step RT-PCR protocol was adopted for all amplifications: 95 °C for 5 min, followed by 42 cycles of 95 °C for 10 s and 60 °C for 30 s, the fluorescence was measured at the end of each cycle. After 42 cycles, a melting curve was acquired by heating the amplicon from 65 to 95 °C to confirm whether a single product was generated. Then, the threshold values (Cq) of each sample were estimated, and the data were exported into Excel format for further primer expression stability analysis. The specificity of the amplification was analyzed through melting curve analysis [71]. All assays were performed with three technical replicates.

To estimate the expression stability of reference genes, Cq values were converted into non-normalized relative quantities, and corrected by PCR efficiency using the formula Q = (E + 1)^−∆Cq^ where E represents the amplification efficiency of each gene, and ∆Cq represents the Cq values minus the lowest Cq value of each gene [52]. These data were imported into two Microsoft Excel-based statistics tools, geNorm [43] and NormFinder [46], which were performed according to the manuals.

### 4.5. Validation of Reference Genes

To confirm the amplification efficiency and adaptability of selected reference genes in different *Morchella* species, ten different morel species including five Esculenta clade species and five Elata clade species were chosen to examine the adaptability of eight ideal reference genes according to the previous results. The strains were identified by multigene phylogenetic analysis (Table 4) [47], whose adaptability was validated by using the same reaction system and protocol for quantitative real-time PCR analysis. After amplification, the PCR products were examined by electrophoresis in a 1.8% agarose gel. One single and clear band in an expected length indicated that the sample can be used as a suitable species adaptability reference gene for qRT-PCR normalization in *Morchella*.

## Figures and Tables

**Table 1:** The candidate internal control genes for gene expression evaluation in this study.

Primer Name	Gene Number ^a^	Gene Description ^b^	*p*-Value ^c^	Primer Sequence	Amplicon Size (bp)	Tm (°C)	E (%)	R^2^	TMP Average ^d^	TMP CV ^e^
GPD2F	TRINITY_DN12699_c0_g1	Glyceraldehyde-3-phosphate dehydrogenase	3.00 × 10^−167^	GGTACGACAACGAATGGGGT	197	60.04	93.8	0.997	405.63	0.2815
GPD2R				CCGCAAAAATCTCTTCCCGC		60.18				
ACT1F	TRINITY_DN11687_c0_g1	Actin, gamma	0	GTACCCTGGTATTGCCGACC	197	60.18	98.8	0.999	746.06	0.1969
ACT1R				GGACGATGGAAGGACCACTC		59.82				
PUB4F	TRINITY_DN12852_c0_g2	Polyubiquitin	9.00 × 10^−158^	GTCCACCCTTCACTTGGTTCT	163	59.86	105.8	0.998	1232.22	0.546
PUB4R				ACGTTGTTGATCTGGGGGAAT		59.65				
CYC3F	TRINITY_DN9811_c0_g1	Peptidyl-prolyl cis-trans isomerase	3.00 × 10^−86^	TCCTCCATCCTCCGAACACA	208	60.25	117.4	0.997	3505.12	0.3792
CYC3R				TCTCAGCGGTTCTAGGGACA		59.96				
ATU2F	TRINITY_DN12747_c0_g4	Alpha-tubulin 2	0	TTCGACGGGCATCTGAATGT	209	59.75	93.3	0.995	17.69	0.4123
ATU2R				ATGTATTTCCCCTTCGCGGG		60.18				
AEF3F	TRINITY_DN10564_c0_g2	Elongation factor 1-alpha	0	CTGTCATTGATGCCCCTGGT	169	60.03	106.7	0.992	6267.16	0.4861
AEF3R				CCAAGGGTGTAGGCGAGAAG		60.11				
HP901F	TRINITY_DN10080_c0_g1	HSP90-domain-containing protein	0	GAGAAGGTTGTCGTCTCGCA	182	60.04	97.8	0.996	418.67	0.1946
HP901R				TTGACGATGGGGGACTTTGG		59.96				
HP701F	TRINITY_DN9777_c0_g1	Hsp70 protein-like protein	1.00 × 10^−119^	TTTGGTGCGTCAGCGTATCT	206	60.04	99.7	0.995	194.33	0.3632
HP701R				GCGGTGCCATTGTGTTTCAT		60.04				
ATU1F	TRINITY_DN5830_c0_g1	Alpha-tubulin	0	TTTCGCTGTGGTGTGGGTAG	169	60.25	91	0.998	422.43	0.335
ATU1R				ATGGAAAACGGAAATGGGAACC		59.43				
BTU1F	TRINITY_DN10333_c0_g2	Beta-tubulin	0	CAGTCGTTCCTTCGCCCAAA	229	60.88	97	0.997	368.32	0.2409
BTU1R				CCAGGGAAACGAAGGGATGT		59.67				
INTF1F	TRINITY_DN10662_c0_g1	Similar to DNA damage response protein wss1	5.00 × 10^−104^	CCGCCAGTCCTAACTCACTC	179	59.83	104.5	0.987	10.35	0.0313
INTF1R				CGATAGCTTTGTCGGGTCCA		59.83				
INTF2F	TRINITY_DN13390_c6_g2	Vesicle transport protein, Got1/SFT2-like	1.00 × 10^−66^	AGATTCCGTAAAAGCGGGGG	238	60.11	104.7	0.994	35.04	0.0355
INTF2R				GCGCATTAACCGAGTGCAAA		60.11				
INTF3F	TRINITY_DN9447_c0_g1	Vacuolar protein sorting/targeting protein 10	0	CTGTTGATGGGTTGGCTGGT	229	60.54	94.4	0.995	25.38	0.0528
INTF3R				GGGTTCATAGGCTTCGTCGT		59.82				
INTF4F	TRINITY_DN8632_c0_g1	Vacuolar ATP synthase 16 kDa proteolipid subunit	2.00 × 10^−57^	CTGCTTTGGTGCCAGTTACG	217	59.76	107.9	0.999	869.67	0.0528
INTF4R				GGAAAGACCAGCACCGAGTT		60.25				
INTF5F	TRINITY_DN12928_c0_g1	Similar to Ubiquitin carboxyl-terminal hydrolase 4	0	TCAGTGTCCCACCCGTATCT	211	59.96	103.3	0.999	16.53	0.0544
INTF5R				CCGCGTGGAGTTATTGGAGT		60.11				
INTF6F	TRINITY_DN12591_c0_g2	Similar to GTPase-activating protein BEM2/IPL2	0	AGAATGGGTTGACGCTGGTT	173	59.89	101.3	0.998	12.15	0.0671
INTF6R				CAGTTGAGGAGGGTTCGCAT		60.04				
INTF7F	TRINITY_DN13203_c1_g1	14-3-3 protein	3.00 × 10^−148^	GCACGTTGGAAAGATCCGTG	170	59.83	99.2	0.995	766.99	0.0725
INTF7R				CGGCGAGATAACGGTGGTAG		60.32				
INTF8F	TRINITY_DN7177_c0_g1	Hypothetical protein	/	GATGCCCGATCCGATGCTC	274	60.74	92	0.989	37.43	0.0713
INTF8R				TGGGCGAGGGTATTTTGGAG		59.75				
INTF9F	TRINITY_DN13335_c6_g2	Select seq ESZ90392.1 splicing factor 3b	0	ACGACACCGACTACGAATCC	239	59.55	92.2	0.987	25.29	0.0733
INTF9R				CGCCTCCAGATACGCACTAA		59.62				

**^a^** Gene number corresponding to the transcriptome data. **^b^** Gene name of the gene nr blast annotation result. ^c^ p-Value of the gene nr blast annotation result. ^d^ Transcripts per million expression in the transcriptome. ^e^ Coefficient of variation of the transcripts per million expression in the different development stages.

**Table 2:** Candidate genes ranked by the expression stability calculated by geNorm.

Ranking	Total		Development Stages		Carbon Resources		Temperature Stress	
	geNorm	Stability Value	geNorm	Stability Value	geNorm	Stability Value	geNorm	Stability Value
1	ACT1	0.54	INTF5	0.23	INTF2	0.23	CYC3	0.19
2	ATU1	0.54	CYC3	0.23	AEF3	0.23	INTF3	0.19
3	INTF4	0.58	PUB4	0.29	INTF7	0.29	ATU1	0.23
4	AEF3	0.61	INTF3	0.34	BTU1	0.32	INTF5	0.26
5	INTF7	0.63	INTF7	0.37	INTF4	0.35	INTF9	0.31
6	INTF5	0.68	INTF6	0.4	INTF9	0.39	INTF7	0.34
7	INTF2	0.76	HP901	0.45	INTF5	0.42	HP901	0.37
8	INTF9	0.8	INTF4	0.48	INTF8	0.44	INTF4	0.42
9	INTF8	0.84	ATU1	0.5	INTF1	0.47	AEF3	0.46
10	ATU2	0.86	INTF9	0.54	ATU1	0.5	ACT1	0.5
11	CYC3	0.89	INTF8	0.57	ACT1	0.52	INTF6	0.55
12	HP901	0.91	INTF1	0.6	HP701	0.56	INTF2	0.58
13	INTF6	0.94	ACT1	0.63	INTF6	0.6	GPD2	0.61
14	PUB4	0.97	INTF2	0.66	INTF3	0.63	BTU1	0.64
15	INTF3	1.02	ATU2	0.7	HP901	0.7	INTF8	0.68
16	INTF1	1.06	HP701	0.74	GPD2	0.77	PUB4	0.73
17	BTU1	1.13	AEF3	0.78	PUB4	0.84	ATU2	0.76
18	HP701	1.21	GPD2	0.91	CYC3	0.95	INTF1	0.82
19	GPD2	1.29	BTU1	1.01	ATU2	1.06	HP701	0.94
Best pair	ACT1/ATU1		INTF5/CYC3		INTF2/AEF3		CYC3/INTF3	

**Table 3:** Candidate genes ranked by the expression stability calculated by NormFinder.

Ranking	Total		Development Stages		Carbon Resources		Temperature Stress	
	Norm Finder	Stability Value	Norm Finder	Stability Value	Norm Finder	Stability Value	Norm Finder	Stability Value
1	INTF7	0.174	INTF5	0.105	INTF4	0.123	ATU1	0.058
2	INTF4	0.254	ATU1	0.178	AEF3	0.157	INTF3	0.058
3	INTF5	0.287	CYC3	0.205	INTF7	0.207	INTF5	0.064
4	AEF3	0.439	INTF7	0.208	INTF2	0.211	CYC3	0.093
5	ATU1	0.439	INTF6	0.222	ACT1	0.247	INTF9	0.185
6	ACT1	0.472	HP901	0.292	ATU1	0.257	INTF7	0.226
7	INTF9	0.472	PUB4	0.32	BTU1	0.26	HP901	0.299
8	INTF2	0.511	INTF3	0.331	INTF8	0.347	INTF4	0.313
9	HP901	0.514	INTF4	0.335	INTF9	0.381	AEF3	0.35
10	INTF8	0.523	INTF1	0.357	HP701	0.426	ACT1	0.42
11	ATU2	0.562	ACT1	0.378	INTF1	0.448	INTF8	0.485
12	INTF6	0.615	INTF9	0.475	INTF5	0.449	INTF2	0.521
13	CYC3	0.632	INTF8	0.524	INTF6	0.631	INTF6	0.536
14	PUB4	0.672	AEF3	0.57	HP901	0.64	GPD2	0.591
15	INTF3	0.787	INTF2	0.614	INTF3	0.671	PUB4	0.603
16	INTF1	0.861	ATU2	0.626	GPD2	0.726	BTU1	0.622
17	BTU1	0.987	HP701	0.776	PUB4	0.85	ATU2	0.659
18	HP701	1.196	GPD2	1.208	CYC3	1.129	INTF1	0.864
19	GPD2	1.325	BTU1	1.248	ATU2	1.264	HP701	1.317
Best pair	INTF7		INTF5		INTF4		ATU1	

**Table 4:** The information of the ten different *Morchella* species used in this paper.

Test Number	Strains Number	Species Name	Phylogenetics Number	Clade
1	Ma_2	*Morchella importuna*	Mel-10	Elata
2	16-9	*Morchella sextelata*	Mel-6	Elata
3	16-41	*Morchella* sp. Mel-21	Mel-21	Elata
4	16-57	*Morchella spongiola*	Mel-21/33	Elata
5	16-50	*Morchella costata*	Mel-13/26	Elata
6	16-72	*Morchella* sp. Mes-23/24	Mes-23/24	Esculenta
7	16-103	*Morchella* Mes-6	Mes-6	Esculenta
8	Win8	*Morchella* sp. Mes-21	Mes-21	Esculenta
9	shanyang	*Morchella* Mes-19	Mes-19	Esculenta
10	M10	*Morchella* sp.	Mes-24	Esculenta
